# Resolving molecule-specific information in dynamic lipid membrane processes with multi-resonant infrared metasurfaces

**DOI:** 10.1038/s41467-018-04594-x

**Published:** 2018-06-04

**Authors:** Daniel Rodrigo, Andreas Tittl, Nadine Ait-Bouziad, Aurelian John-Herpin, Odeta Limaj, Christopher Kelly, Daehan Yoo, Nathan J. Wittenberg, Sang-Hyun Oh, Hilal A. Lashuel, Hatice Altug

**Affiliations:** 1 Institute of Bioengineering, École Polytechnique Fédérale de Lausanne (EPFL), 1015 Lausanne, Switzerland; 2 ICFO – Institut de Ciències Fotòniques, The Barcelona Institute of Science and Technology, 08860 Castelldefels, Spain; 3 Laboratory of Molecular and Chemical Biology of Neurodegeneration, Brain Mind Institute, School of Life Sciences, École Polytechnique Fédérale de Lausanne (EPFL), 1015 Lausanne, Switzerland; 4 School of Chemistry, University of Glasgow, Joseph Black Building, Glasgow, G12 8QQ UK; 5 Department of Electrical and Computer Engineering, University of Minnesota, Minneapolis, MN 55455 USA; 6 Department of Chemistry, Lehigh University, Bethlehem, PA 18015 USA

## Abstract

A multitude of biological processes are enabled by complex interactions between lipid membranes and proteins. To understand such dynamic processes, it is crucial to differentiate the constituent biomolecular species and track their individual time evolution without invasive labels. Here, we present a label-free mid-infrared biosensor capable of distinguishing multiple analytes in heterogeneous biological samples with high sensitivity. Our technology leverages a multi-resonant metasurface to simultaneously enhance the different vibrational fingerprints of multiple biomolecules. By providing up to 1000-fold near-field intensity enhancement over both amide and methylene bands, our sensor resolves the interactions of lipid membranes with different polypeptides in real time. Significantly, we demonstrate that our label-free chemically specific sensor can analyze peptide-induced neurotransmitter cargo release from synaptic vesicle mimics. Our sensor opens up exciting possibilities for gaining new insights into biological processes such as signaling or transport in basic research as well as provides a valuable toolkit for bioanalytical and pharmaceutical applications.

## Abstract (web summary)

Complex protein-lipid interactions are difficult to study in real-time without labels. Here, Rodrigo *et al*. introduce a multimodal plasmonic infrared biosensor to simultaneously distinguish multiple analytes with high sensitivity.

## Introduction

Optical spectroscopy approaches, including infrared (IR) absorption, circular dichroism (CD), and Raman scattering, are powerful label-free techniques for detecting biomolecules and extracting detailed chemical information in a real-time and non-destructive manner^1–4^. The mid-infrared spectral range is of pivotal importance for these detection approaches due to the presence of strong characteristic absorption fingerprints, which enable the specific identification of biomolecules of different chemical nature^5–10^. Importantly, all basic building blocks of life such as lipids, proteins, and nucleic acids exhibit distinct and unique fingerprints in this spectral range. Detecting association, dissociation, and other molecular interactions in mixtures of these components are crucial for understanding a multitude of biological systems and processes in health and disease. Consequently, differentiating and monitoring individual components in such heterogeneous mixtures are a central objective in biosensing. It is particularly important for the understanding of pathological conditions, where uncontrolled interactions of cellular membranes with lytic peptides lead to membrane perforation and cell death^11,12^. Resolving multi-analyte systems are also crucial in studying lipid vesicles encapsulating cargo molecules, which are utilized by most cell types as biological shuttles and regulate a multitude of important cellular processes^13^. For instance, synaptic vesicles in neurons loaded with neurotransmitter molecules can release their cargo upon stimulus to transmit chemical signals to postsynaptic neurons, thereby making perception and thought possible^14^.

Resolving such dynamic membrane processes with standard label-free assay techniques such as quartz crystal microbalance (QCM)^15^ and surface plasmon resonance (SPR)^16^ is extremely challenging. Indeed, the competing effects of protein association, lipid membrane perforation, and cargo release on net analyte mass and refractive index make it difficult to decouple the contribution of the individual analytes from the overall signal. Therefore, to study complex interactions in multi-analyte biological systems, it is pivotal to develop new label-free sensing platforms that can chemically discriminate different biomolecular species and independently track their time evolution.

Infrared absorption spectroscopy is the prime tool for addressing such challenges, but traditional implementations struggle in resolving submonolayer films and ultrathin membrane systems. Metasurfaces excel at controlling light on the nanoscale and provide a powerful platform for tailoring the spectral response and light localization in nanophotonic devices^17–20^. Engineered metasurfaces can create intense highly confined hot spots of the electromagnetic field, providing strong interaction with adjacent analytes and thus making them ideal candidates for biosensing and spectroscopy applications^21–27^. Furthermore, the vibrational–absorption enhancement of IR metasurfaces extends tens of nanometers from their surface^28^, making them suitable for multi-layer assays which are not accessible by their Raman counterparts (surface-enhanced Raman spectroscopy), whose enhancement is limited to only a few nanometers^29,30^.

In this work, we present a chemically specific, label-free nanophotonic biosensor for distinguishing multiple analytes in dynamic lipid membrane processes. Our infrared sensor uses a multi-resonant metasurface consisting of self-similar overlapping nanoantenna arrays to provide up to 1000-fold near-field intensity enhancement over multiple spectral bands. We exploit this metasurface sensor concept to unravel the interaction of biomimetic lipid membranes with different polypeptides as well as the dynamics of vesicular cargo release. Real-time, chemical-specific detection of these biological entities is performed by extracting their characteristic fingerprints in the amide and methylene absorption regions. Significantly, we leverage our technology to study the interaction of lipid membrane systems with melittin, a toxic pore-forming peptide. We show that our technique can monitor in real time the melittin association process and the simultaneous disruption of the lipid membrane with high sensitivity, exquisite chemical specificity and without labeling. Extending this concept to more complex dynamic membrane processes, we then leverage our method to monitor melittin-induced neurotransmitter cargo release from synaptic vesicle mimics, paving the way toward exciting applications in neurobiology and drug development for brain-related diseases.

## Results

### Multi-resonant metasurface for molecule-specific detection

**Fig. 1:**
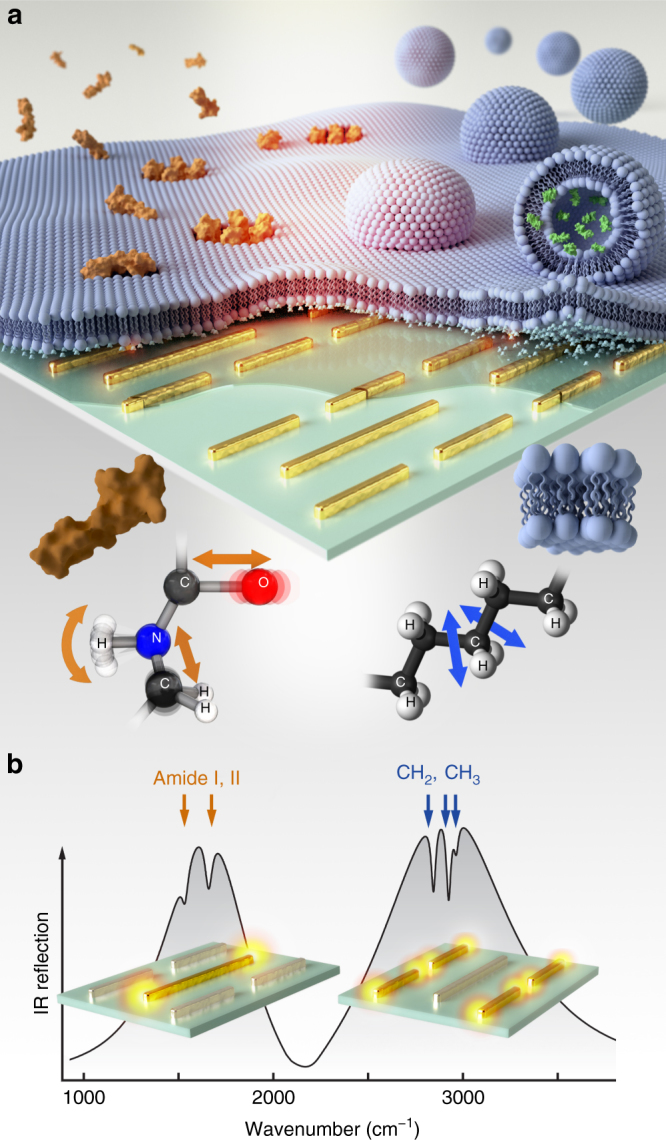
Nanophotonic label-free biosensor for chemically distinguishing multiple analytes in biological samples. **a** Multi-resonant mid-IR nanoantennas are leveraged to enhance the vibrational–absorption signals associated with biomimetic lipid membrane formation, polypeptide/membrane interaction, and vesicular cargo release on the sensor surface. **b** Antenna resonance positions are engineered to simultaneously overlap with the vibrational signatures of both the amide I, II, and the CH_2_, CH_3_ absorption bands, allowing for the simultaneous enhancement and detection of lipid- and protein-induced absorption changes. The 3D model of melittin used in this figure was imported from RSCB Protein Data Bank, DOI: 10.2210/pdb2MLT/pdb, which was deposited by D. Eisenberg, M. Gribskov, and T.C. Terwilliger. All rights reserved

The strong near fields excited in the vicinity of metallic nanoantennas provide an ideal platform to enhance the weak vibrational fingerprint signals originating from nanometer-sized analytes and biological membranes^31^. Typically, the resonance frequency of mid-IR nanoantennas is spectrally tuned to overlap with the characteristic vibrational modes of the target biomolecules, which has previously been demonstrated using arrays incorporating a single antenna length^6,28^. Extending this functionality to multi-analyte biosensing requires a metasurface that supports multiple resonances that can be individually tuned to match the characteristic vibrations of the different analytes of interest (Fig. 1). Current approaches mainly use a uniform array of multi-resonant elements^23,24^. However, resonances in such systems are typically excited with reduced efficiency levels for higher-order modes associated with smaller resonant feature sizes, resulting in decreased surface-enhanced infrared absorption (SEIRA) performance at these frequencies^32–35^. Furthermore, the electromagnetic coupling between the different resonant modes impedes a straightforward spectral tuning of the individual resonance frequencies^33,35^. Our approach overcomes these two major limitations by allocating distinct resonances on multiple overlapping arrays with different periodicities.

**Fig. 2:**
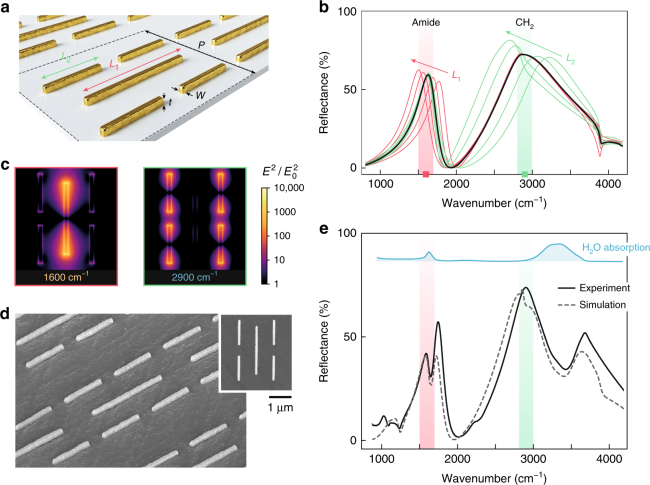
Multi-resonant metasurface sensor platform. **a** Schematic of the multi-resonant mid-IR metasurface composed of two sets of gold nanodipoles (*L*_1 _= 1.8 µm, *L*_2 _= 0.95 µm, *P* *=* 2.6 µm, *W* *=* *t* *=* 100 nm). **b** Simulated reflectance spectrum of the multi-resonant metasurface for the nominal design (black curve), and with varying lengths *L*_1_ (red curves) and *L*_2_ (green curves) in a ±10% range. An immersion media with refractive index *n* = 1.32 has been considered to represent the aqueous environment. The two resonances are independently adjusted to overlap with amide and CH_2_ bands. **c** Near-field distribution of the multi-resonant metasurface parallel to the substrate plane at the amide and CH_2_ bands. Each set of dipole nanoantennas is excited and exhibits strong near fields (bright yellow color) only for the corresponding resonance frequency. **d** Scanning electron microscope image of the nanofabricated multi-resonant metasurface. **e** Experimental reflectance spectra of the multi-resonant metasurface in phosphate buffer saline (PBS) solution. The full frequency–dispersive complex refractive index of water has been considered in the simulated reflectance spectrum^36^. Peak positions agree well with the simulations from (**b**). The additional dips in the peak lineshapes are due to the absorption bands of water in the mid-IR (blue-shaded area)

Our metasurface design is composed of two sets of gold nanodipoles that provide simultaneously a low- and high-frequency resonance, which can be individually adjusted by tuning the corresponding dipole lengths *L*_1_ and *L*_2_ (Fig. 2a). The lengths are designed to be approximately half the corresponding operating wavelength accounting for the dielectric environment. Additionally, the periodicity is adjusted to make the accumulated scattering cross-section of the dipoles equal to the geometrical cross-section of the array, resulting in high reflectance values between 60 and 75% at resonance. In particular, this requires the density of short dipoles (length *L*_1_) in the multi-resonant metasurface to be four times larger than that of long dipoles (length *L*_2_). This design concept can also be understood as overlapping dipole arrays, where the lateral dimensions (both the dipole length and periodicity) of the second array have been almost equally scaled with respect to the first array. Because of this self-similar geometry, the spectral response is composed of two resonances that are spectrally separated by the geometric scaling factor between arrays and are excited with almost identical efficiency. Importantly, our versatile multi-periodicity self-similarity concept is scalable to achieve multi-resonant devices with more than two peaks, if required by the target application (Supplementary Fig. 1).

The optical response of the multi-resonant array is investigated using a 3D Maxwell equation solver based on the finite element method (see Methods). The simulated reflectance spectrum of the nanoantenna array contains two well-defined resonances as shown in Fig. 2b and Supplementary Fig. 2. These resonances are carefully adjusted to spectrally match with the amide and CH_2_ spectral regions, which contain the intense IR absorption bands of proteins and lipids, respectively. Specifically, we target the amide I and II bands located around 1560 and 1660 cm^−1^ as well as the methylene doublet located around 2850 and 2930 cm^−1^. Naturally, in addition to proteins and lipids, peptides and other chemicals can equally be detected with our method as long as they support distinct fingerprint signatures within the resonance bands of the metasurface. Modifying *L*_1_ or *L*_2_ shifts the corresponding resonance while leaving the other resonance unaffected, indicating that the two sets of nanodipoles are weakly coupled and their electromagnetic response is independent from each other. Such independence is a key characteristic that allows the metasurface design to be adjusted in a straightforward manner to enhance selected vibrational bands from different analytes.

Simulated near-field distributions (Fig. 2c) show that only one set of nanodipoles is resonantly excited for each spectral band of interest, which indicates that our metasurface design can also provide spatial sensitivity by detecting only the molecules placed in the near-field hot spots of the corresponding antennas. Even though this capability is not exploited in this work, it could find applications in multiplexed detection and imaging-based techniques. On the other hand, the spatial sensitivity can be a disadvantage in experiments where the analytes are inhomogeneously distributed over the surface, or if it is required that the vibrational signals arise from the same molecules for all the utilized infrared bands. Overall, our design provides maximum local near-field intensity enhancements between three and four orders of magnitude for both bands. Furthermore, the near-field intensity extends up to tens of nanometers from the metasurface (Supplementary Fig. 3), providing penetration depths which are comparable with those in state-of-the-art single-band SEIRA substrates. Such extended penetration depths are in contrast to the 1–2 nm depths achieved by surface-enhanced Raman spectroscopy and gives SEIRA a unique advantage for probing lipid membranes and vesicles.

The multi-resonant array is fabricated on an IR-transparent CaF_2_ substrate by electron beam lithography and a lift-off process (see Methods). Scanning electron microscope images of the device are shown in Fig. 2d. The IR reflection spectrum of the array is measured with a Fourier Transform IR (FTIR) spectrometer, illuminating the chip from the backside of the substrate and immersing it in phosphate-buffered saline (PBS). The measured reflectance spectrum shown in Fig. 2e is consistent with the dual-resonance spectrum shown in Fig. 2b when the IR absorption bands of water are incorporated and is in good agreement with electromagnetic simulations accounting for the frequency–dispersive complex refractive index of water^36^ (also see Supplementary Note 2). More importantly, the measured results confirm the multi-resonant response of the metasurface, providing near-field enhancements at two spectral bands that overlap with the amide and CH_2_ vibrations, making it an excellent candidate for multi-analyte mid-IR biosensing.

### Simultaneous monitoring of multiple analytes

To demonstrate the capability of monitoring and distinguishing multiple biological analytes simultaneously, we use a bioassay based on the additive association between streptavidin (SA) and a phospholipid membrane. Specifically, we utilize 1,2-dioleoyl-sn-glycero-3-phosphocholine (DOPC) vesicles to form a supported lipid bilayer (SLB) on the sensor surface, which acts as a model cell membrane for the study of protein interaction kinetics^37^. Prior to experiments, our plasmonic sensor chips are functionalized with a 10 nm silicon dioxide layer coated by atomic layer deposition and exposed to a short oxygen plasma treatment to provide a hydrophilic surface suitable for membrane formation^28,38–41^. Bilayer membrane fluidity and thickness are confirmed via fluorescence recovery after photobleaching (FRAP) and surface plasmon resonance (SPR) experiments (Supplementary Fig. 4 and 5). To selectively bind SA to the membrane, vesicles for bilayer formation are prepared from a lipid mixture consisting of DOPC and a small percentage of biotinylated lipids.

**Fig. 3:**
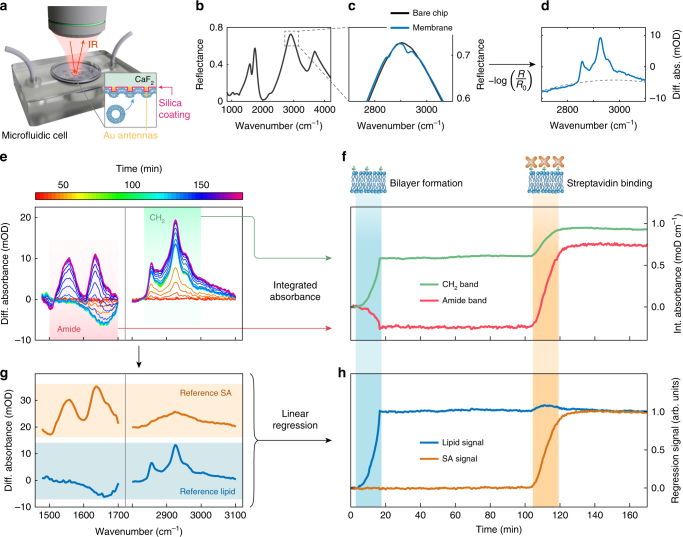
Simultaneous monitoring of multiple biological analytes. **a** Schematic of the experimental configuration. **b** Infrared reflectance spectrum of the multi-resonant sensor chip in PBS buffer solution. **c** Reflectance spectra before (*R*_0_) and after (*R*) lipid membrane formation in the CH_2_ band spectral region, magnified from marked area in (**b**). **d** Differential absorption spectrum calculated from the reflectance spectra in (**c**). The dashed line corresponds to the second-order polynomial used for baseline correction. **e** Color-coded time-dependent differential absorption spectra acquired during the lipid membrane formation and streptavidin-binding experiment. **f** Time trace of the integrated absorbance signal in the amide (red-shaded area) and CH_2_ (green-shaded area) bands from (**e**). The lipid and streptadivin injection steps are indicated by the blue- and orange-shaded areas, respectively. The integrated absorbance signals from the amide (red curve) and CH_2_ (green curve) bands exhibit pronounced signal modulations during the lipid membrane formation and streptavidin-binding steps, evidencing an inadequate discrimination of the two analytes. **g** Reference spectra for the lipid (blue-shaded area) and streptavidin (orange-shaded area) signal contributions. **h** Linear regression signals obtained from the spectral data in (**e**) with respect to the reference spectra in (**g**). Linear regression signals for lipid (blue curve) and streptadivin (orange curve) show a significant signal increase only during the corresponding lipid or streptavidin injection step, demonstrating effective chemical discrimination

During biosensing measurements, the multi-resonant metasurface chip is placed into a polydimethylsiloxane (PDMS) microfluidic cell to allow for the controlled delivery of the various molecules (Fig. 3a). Real-time mid-IR reflectance spectra are measured with an FTIR spectrometer from the backside of the metasurface to prevent the complete absorption of infrared light by water and enable in-solution mid-IR experiments. This integrated microfluidic approach allows us to resolve the time evolution of both the lipid bilayer formation and subsequent streptavidin-binding kinetics in situ. A typical reflectance spectrum of the metasurface chip immersed in PBS buffer solution is shown in Fig. 3b. In addition, a magnified view of the reflectance in the CH_2_ spectral region is presented in Fig. 3c, highlighting the absorption fingerprint after lipid membrane formation on the sensor chip. To aid the subsequent analysis of the time-resolved spectral data, the reflectance spectra are converted to differential absorption (Fig. 3d) and baseline corrected^28,42^.

The time evolution of the differential absorbance spectra over the course of the experiment is shown in Fig. 3e for both amide and CH_2_ bands. After filling the fluidic channels with PBS buffer solution, 100 nm diameter DOPC vesicles containing 5% of biotin-functionalized lipids were injected and allowed to form the SLB. Subsequently, streptavidin was injected to bind to the biotinylated lipids present in the membrane (see Methods). The onset of absorption signals in both bands can clearly be observed in the differential absorbance results, indicating successful lipid membrane formation and streptavidin association in the bioassay. To further visualize these results, we calculate and trace the integrated absorbance from 1500 to 1700 cm^−1^ (amide I–II) and from 2800 to 3000 cm^−1^ (CH_2_) over time (Fig. 3f). The lipid bilayer formation results in a pronounced increase of the integrated absorbance in the CH_2_ bands, while streptavidin binding produces a respective increase in the amide-integrated absorbance. However, since the molecular structure of streptavidin contains CH_2_ groups, the streptavidin injection also produces a change in the CH_2_ signal. A similar effect can be observed in the amide-integrated absorbance during lipid membrane formation, where water displacement produces a negative absorbance signature due to the H_2_O in-plane bending mode. This complex behavior indicates that a simple tracking of the integrated absorbance is insufficient to resolve the different analytes in multi-component systems, especially if the constituent biomolecules have overlapping absorption signatures.

To overcome this challenge, we perform linear regression of the time-dependent differential absorbance data with respect to reference spectra for DOPC lipids and streptavidin (Fig. 3g). Reference spectra were obtained by measuring an SLB for the lipid reference and, independently, a protein monolayer formed by physisorption for the streptadivin reference. The lipid and protein signals directly correspond to the coefficients in the linear combination of reference spectra that minimize deviation from the measured reflectance spectrum under the least square criterion (see Methods). This approach enables us to separate and identify the contributions of the two biological components in the full differential absorbance signal (Fig. 3h)^43^. The effective chemical discrimination of the components becomes obvious when examining the lipid and streptavidin regression signals, which show a significant signal increase only during the corresponding lipid and streptavidin injection steps, respectively. The extracted values of the regression signals correspond to the analyte mass on the sensor surface relative to the amount present in the corresponding reference. As a result, when the surface density of the molecules changes, the regression signals vary accordingly (Supplementary Fig. 6).

To confirm that two reference spectra used are sufficient to capture the full biological information in our time-dependent measurements, we performed principal component analysis (PCA) over the spectral data corresponding to all of the time points, and found that 99.7% of the total variance is accounted for by the first two principal components (Supplementary Fig. 7). Importantly, our platform is not limited to two biochemical species and can be extended to bioassays with a higher number of biological components by including the corresponding reference spectra in the linear regression analysis, as will be shown in the next section. On the other hand, the linear regression approach has limitations when exploring unknown samples or analytes whose absorption spectra are not known. A PCA approach could be more suitable in these cases, but the interpretation based on principal component scores would be significantly more challenging (Supplementary Note 7).

### Melittin-induced pore formation in membranes

Moving beyond simple additive protein–membrane association processes, we investigated more complex dynamics of lipid membrane disruption induced by toxic peptides. For this purpose, we utilize melittin, a hemolytic peptide constituting the main toxic component of apitoxin, the bee venom^44^. Melittin is well known for its efficient association with lipids and the resulting perforation of lipid membranes through the formation of pores^45^. Due to its cytotoxicity and membrane disruptive properties, melittin is a naturally occurring anti-microbial and holds potential for the treatment of immune-related diseases such as many types of cancer^46^. To demonstrate the versatility of our approach, we study melittin-induced pore formation in membranes for two distinct biological schemes: the disruption of a supported lipid membrane and the release of encapsulated cargo from surface-attached lipid vesicles.

**Fig. 4:**
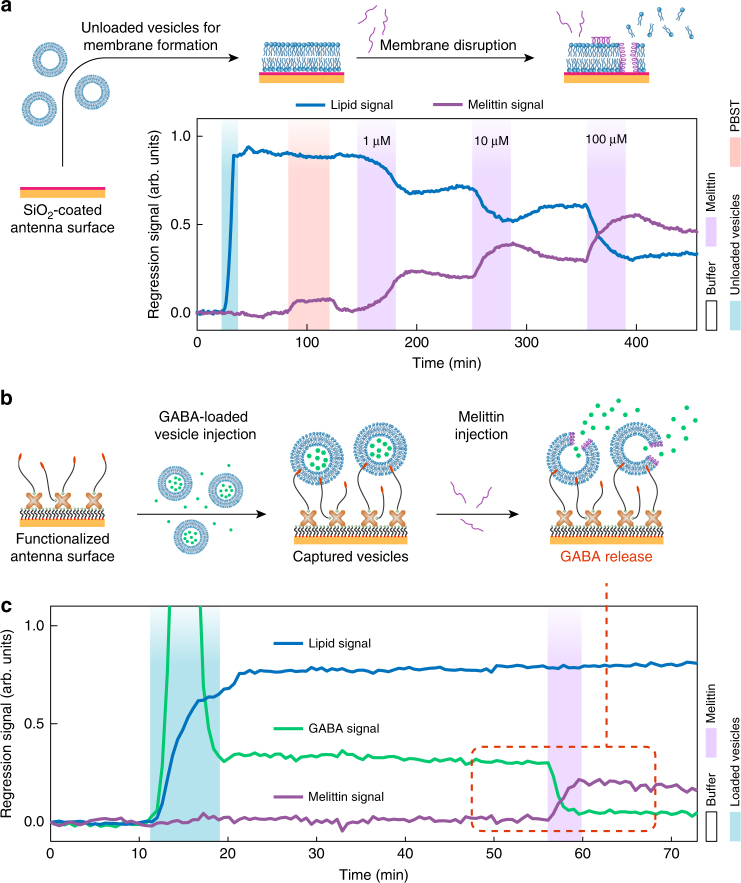
Melittin-induced membrane disruption and vesicular cargo release. **a** Melittin association to the supported lipid bilayer (SLB) and melittin-induced disruption of the membrane for increasing melittin concentrations (1, 10, and 100 µM). The time evolution of the melittin linear regression signal (purple) shows melittin-membrane association and partial dissociation phases for each melittin injection time step. The increase in melittin signal is accompanied by a clear decrease in the lipid regression signal (blue) evidencing loss-of-membrane integrity, which intensifies with increasing melittin concentrations. **b** Sketch of the vesicle cargo release experiment. The sensor metasurface is functionalized with hydrophilic tethers displaying cholesterol moieties, which are then used to capture lipid vesicles loaded with the neurotransmitter gamma-aminobutyric acid (GABA). Injection of melittin perforates the lipid vesicle membrane, resulting in a release of GABA cargo molecules. **c** Time-resolved linear regression signals for the three characteristic biological components in the experiment: lipid, GABA, and melittin. After the injection of GABA-loaded vesicles, successful attachment of intact, loaded vesicles to the surface is corroborated by the stable lipid and GABA regression signals. The strong initial peak of the GABA signal is caused by the transient flow of extravesicular GABA molecules present in the bulk solution. Melittin injection results in a fast and pronounced decrease of the GABA signal, indicating efficient cargo release

In the first scheme, we monitor the interaction between lipids and melittin by forming an SLB from unloaded DOPC vesicles, next removing residual vesicles with PBST and subsequently injecting increasing concentrations of melittin (Fig. 4a). In contrast to the previous additive association measurements, we observe a clear decrease of the lipid signal upon melittin injection, which correlates with a strong increase of the melittin signal after a transient fluctuation induced by PBST. This behavior is caused by insertion of melittin into the lipid membrane, which leads to the displacement of lipids due to the formation of nanosized pores^47^. The observed strong signal modulation is caused by the efficient association of melittin to the DOPC lipids, which is confirmed with independent bulk circular dichroism measurements (Supplementary Fig. 8). Increasing the injected melittin concentration from 1 to 100 µM demonstrates a progressively higher and non-proportional perforation of the lipid membrane. Particularly, for the highest concentration of 100 µM, more than 60% of the lipid molecules are displaced from the surface based on the lipid regression signal, which is also corroborated by independent fluorescence experiments (Supplementary Fig. 9). The recorded signals capture clearly the melittin association and dissociation during melittin injection and PBS rinsing steps, respectively. The association kinetics for 1 µM concentration are qualitatively different than for 10 and 100 µM, which we attribute to the presence of melittin molecules in the membrane after the first injection step (1 µM). During the dissociation phase, we observe that ~80–90% of the adsorbed melittin remains in the membrane after the PBS rinsing, indicating high membrane/melittin affinity. It is important to note that we are able to investigate melittin-binding kinetics on a supported lipid membrane (Supplementary Fig. 5), using melittin concentrations in the same range as in previous works^45^.

This experiment evidences that our sensor platform could go beyond the capabilities of other label-free biosensing approaches based on mass or refractive index detection, such as quartz crystal microbalance (QCM) or surface plasmon resonance (SPR) techniques, which are considered the gold standard in real-time label-free detection of biomolecular interactions^16,48^. These techniques enable the study of binding kinetics in processes where multiple analytes provide a net contribution to the total mass accumulated on the sensor, however they struggle in situations where the injection of one kind of analyte triggers the removal of another analyte that is already present on the surface. The chemical specificity of our technique overcomes this fundamental limitation and provides exciting opportunities for the study of multi-analyte systems.

As a final demonstration, we applied our approach to a system with increased complexity, featuring small analyte molecules in addition to lipid vesicles and peptides. This system exemplifies that our technology can be extended to monitor multiple analytes in more complex scenarios, such as the release of cargo molecules from vesicles in our case. We focus on cholesterol-enriched lipid vesicles containing neurotransmitters, a system mimicking synaptic vesicles naturally found in neurons. Specifically, vesicles are loaded with GABA, which is the major neurotransmitter for inhibitory synaptic transmission. Consequently, GABA uptake and release processes influence a multitude of brain-related diseases and GABA receptors are major drug targets for such illnesses^49^.

To enable the observation of the biological process schematized in Fig. 4b, we first optimized a surface functionalization protocol for capturing intact cargo-filled lipid vesicles with a diameter of 50–70 nm on our metasurface chip. This is achieved by functionalizing the gold antenna surface using biotinylated thiols, followed by the attachment of streptavidin, which is then utilized to bind biotin-PEG-cholesterol vesicle tethers (see Methods). In a second step, GABA-filled vesicles are captured on the functionalized metasurface and the release of the cargo is triggered via the melittin-induced perforation of the vesicle membrane.

To detect the vibrational signature of the GABA molecules, we focus on its distinct absorption peak at 1562 cm^−1^, resulting from the asymmetric stretching of its carboxylate group. Our platform can efficiently detect the absorption peak of GABA via the first resonance mode of the metasurface. Crucially, the GABA absorption signature exhibits strong spectral overlap with the amide II signature of melittin. Therefore, simple analysis based on integrated absorbance over the amide I–II range is unable to simultaneously trace the signals of GABA and melittin. However, since the absorption spectra of the three analytes (lipid, melittin, and GABA) form a linearly independent set (Supplementary Fig. 10), our linear regression approach can easily extract the signal contributions of each molecule. As demonstrated in Fig. 4c, time-resolved linear regression signals for the three biomolecular components in the experiment (lipid, melittin, and GABA) are efficiently distinguished. This result demonstrates the applicability of our approach in biological systems with more than two analytes, as long as their infrared spectra are sufficiently different and linearly independent over the detection bands of the metasurface.

The injection of GABA-loaded vesicles induces a pronounced increase in the signals corresponding to both lipid and GABA channels. After the initial binding phase, both signals remain stable for more than 30 min under continuous buffer solution flow, indicating the capture of intact vesicles on the sensor metasurface. The strong, transient peak of the GABA signal during injection is attributed to extravesicular GABA molecules in the bulk solution, which are subsequently washed away by the flow of buffer solution. Crucially, GABA molecules cannot attach to the functionalized metasurface (Supplementary Fig. 11), confirming that the stable GABA signal in Fig. 4c originates from encapsulated molecules in the vesicles.

After melittin injection, the association of this peptide with the vesicle membrane is clearly detected as an increase of the melittin regression signal. In contrast to our previous SLB experiments, here the lipid regression signal remains constant during melittin association, which is attributed to the accommodation of the inserted melittin molecules in the tethered vesicles via a slight increase of their size, leaving the density of lipid molecules mostly unaffected. On the other hand, the binding of melittin to SLBs in previous experiments produced a lateral displacement of lipid molecules, which lead to the reduction of the lipid surface density (compare to Fig. 4a). Strikingly, the injection of melittin causes a simultaneous and pronounced decrease of the GABA regression signal. This is unequivocal evidence of the melittin-induced cargo release of the vesicles. Based on the relative GABA signal levels before and after the melittin injection step, we conclude that around 85% of the encapsulated GABA molecules are released through the perforated membranes.

## Discussion

We have developed a label-free and chemically specific nanophotonic biosensor for extracting and distinguishing molecule-specific information in multi-analyte biological systems. Our approach leverages a multi-resonant mid-IR metasurface that provides up to three orders of magnitude local near-field intensity enhancements simultaneously over the amide and methylene bands. The introduced metasurface concept is flexible to add additional bands that can be individually adjusted to suit different applications. The combination of real-time spectral acquisition with advanced linear regression analysis allows to discriminate the different analytes and accurately trace the interaction kinetics. We demonstrate that our sensor is well adapted for real-time monitoring of lipid–protein systems in aqueous environments and the study of a range of important processes such as lipid–protein association, protein-induced disruption of membranes and vesicular cargo release.

By studying the interaction of the pore-forming toxin melittin with lipid membranes, we showed that our method could independently trace melittin and lipid signals and reveal melittin-induced disruption of the membrane. This experiment highlights that our platform can greatly contribute to elucidate the underlying mechanisms of anti-microbial, cytolytic, and cell-penetrating peptides. Furthermore, the label-free real-time monitoring of neurotransmitter cargo release from synaptic vesicle mimics demonstrates the applicability of the method for biomolecular systems with increased complexity. In this regard, our sensor can contribute to study important classes of lipid vesicles such as synaptic vesicles in neurodegenerative diseases, exosomes in cancer, as well as drug release mechanisms from liposomes in pharmaceutical research.

## Methods

### Sensor nanofabrication

Metasurfaces are nanofabricated on a CaF_2_ substrate by electron beam lithography and a lift-off process. Poly(methyl methacrylate) (PMMA) is used as the pattern defining electron beam resist and a lower molecular weight sublayer is used to help the lift-off process. The resist is coated with a 5–10 nm-thick gold layer to reduce electron charging during exposure. Metasurfaces with a lateral size of 280 × 280 µm^2^ are exposed with a 100 keV electron beam, developed in MiBK:IPA 1:3 solution and the gold conduction layer is wet etched in KI + I_2_. Metal nanoantennas are formed by evaporation of a 5 nm-thick Cr adhesion layer and a 100 nm-thick Au layer, followed by a lift-off process carried out in acetone. The chips used for experiments with SLBs are then conformally coated with a thin SiO_2_ layer deposited by atomic layer deposition (ALD). The ALD process is performed at 100 °C with alternating cycles of sequentially injecting trimethylaluminum (Al(CH_3_)_3_) and tris(tert-butoxy)silanol ((tBuO)_3_SiOH) with a deposition rate of 1.67 Å per cycle. The gold antennas without a SiO_2_ layer used for cargo release experiments were functionalized ex situ by incubating the chip with 1.5 mM HS-C11-EG3-Biotin (ProChimia Surfaces) in ethanol for at least 12 h.

### FTIR measurements

Infrared spectral measurements are carried out using a Fourier transform infrared (FTIR) spectrometer (Bruker Vertex) coupled to an IR microscope (Hyperion 3000) equipped with a reflective Cassegrain objective (NA = 0.4, ×15) and a mercury cadmium telluride (MCT) detector. The light polarization is applied parallel to the axis of the nanoantennas and the collected light is limited to a 200 × 200 µm^2^ area by knife edge apertures. Experiments are carried out in a dry air purged environment. The measurements are performed in reflectance mode and illuminating the nanoantenna arrays from the backside of the substrate to avoid light propagation across the infrared-opaque water.

For real-time microfluidic measurements, the chip is mounted in a custom-built polydimethylsiloxane (PDMS) microfluidic cell, which is connected to a pump to control the analyte flow through the cell. During experiments with SLBs, the flow rate is fixed at 30 µL min^−1^ apart from the bilayer formation step, which is carried out at a flow rate of 15 µL min^−1^ as shown previously^28^. During the cargo release experiments, the flow rate was 50 µL min^−1^. All experiments are carried out in PBS buffer (10 mM phosphate buffer, 2.7 mM potassium chloride, and 137 mM sodium chloride, pH 7.4). Analyte injections (lipid, streptavidin, melittin, and GABA) are performed using a medium pressure injection valve (Upchurch Scientific V-451) to ensure uninterrupted analyte delivery. For the cargo release experiments, the gold antennas previously modified with biotinylated alkanethiols are further functionalized in situ by flowing 3 μM streptavidin (Thermo Fisher Scientific) followed by 9 μM Cholesterol PEG Biotin (Nanocs Inc.).

### Lipid vesicle experiments

Small unilamellar vesicles are prepared similarly to the protocol from ref. ^50^. Defined ratios of either DOPC (1,2-dioleoyl-sn-glycero-3-phosphocholine, Avanti Polar Lipids, Inc.) and Biotinyl PE (1,2-dipalmitoyl-sn-glycero-3-phosphoethanolamine-*N*-(biotinyl), Avanti Polar Lipids, Inc.) or POPC (1-palmitoyl-2-oleoyl-sn-glycero-3-phosphocholine, Avanti Polar Lipids, Inc.) and cholesterol (Sigma-Aldrich) are mixed in chloroform in a round bottom flask and dried under a stream of N_2_. Remaining solvent is removed by keeping the mixture under vacuum overnight. The dried lipids are resuspended in PBS by vortexing to achieve a 1 mg mL^−1^ solution of lipids, which is then exposed to bath sonication for 30 min. For cargo release experiments, the dried lipids are resuspended in PBS with 100 mg mL^−1^ GABA. After sonication, the solution is then extruded for a minimum of 18 times through 100 nm pore-size polycarbonate filters using an Avestin LiposoFast extruder (Avestin Inc.). After SLB formation in real-time infrared experiments, residual vesicles in the microfluidic system are rinsed with PBS solution containing 0.05% (wt/vol) polysorbate 20 (Tween 20, Sigma-Aldrich) surfactant (PBST). For cargo release experiments, a twofold dilution of the vesicles (20% cholesterol, 80% POPC) in PBS was carried out prior injection and membrane perforation was performed with 2 µM melittin (GenScript).

### FTIR data analysis

The extraction of the IR fingerprints of the analyte is performed by normalizing the reflectance spectra of the sensor to that of the bare sensor before analyte injection. The differential absorbance spectra is calculated by subtracting a second-order polynomial fitted by least-squares method to the normalized reflectance. The integrated absorbance signal is calculated by integrating the extracted amide and CH bands over the 1.525–1.650 cm^−1^ and 2.835–2.935 cm^−1^ ranges, respectively. The protein and lipid signals are obtained by linear least-squares regression of the absorbance spectrum of the heterogeneous sample using as basis the individual reference spectrum of the protein and lipid. The protein and lipid signals (s_P_, s_L_) are calculated as (*s*_P_, *s*_L_) = (*X*^T^*X*)^−1^ *X*^T^ *a*, where *a* is the absorbance spectrum of the heterogeneous analyte and *X* = (*a*_P_, *a*_L_) is the reference absorbance matrix formed by the reference absorbance spectra of the protein and lipid. The reference spectrum for each individual analyte is obtained from independent experiment by measuring: a lipid bilayer formed by vesicle rupture, a streptavidin layer by physisorption, a mellitin layer formed by physisorption and GABA molecules by high concentration (100 mg mL^−1^) in flow measurements.

### Numerical simulations

The spectral and near-field characteristics of the nanoantenna arrays are calculated using a commercial solver of Maxwell equations (Ansys HFSS) based on the finite elements method. The periodicity of the arrays is modeled by periodic boundary conditions delimiting the array unit cell. The structure is excited by an electromagnetic plane wave with light polarization aligned with the dipole antennas and including all propagating Floquet modes. The scattering properties are calculated over an iteratively refined mesh until convergence is reached.

### Data availability

The authors declare that the main data supporting the findings of this study are available within the article and its Supplementary Information files. Extra data are available from the corresponding author upon reasonable request.

## Electronic supplementary material

Supplementary Information
