# Supplementary material for: Resolving molecule-specific information in dynamic lipid membrane processes with multi-resonant infrared metasurfaces
Source: Nat Commun. 2018 Jun 4;9:2160. doi: 10.1038/s41467-018-04594-x (PMC5986821; doi:10.1038/s41467-018-04594-x)
Supplement: Supplementary file 1 — Supplementary Information [file 41467_2018_4594_MOESM1_ESM.pdf]

Supplementary information

Resolving molecule-specific information in dynamic lipid membrane processes with multi-resonant infrared metasurfaces

Daniel Rodrigo[1,2,§], Andreas Tittl[1,§], Nadine Ait-Bouziad[3], Aurelian John-Herpin[1], Odeta Limaj[1], Christopher Kelly[1,4], Daehan Yoo[5], Nathan J. Wittenberg[5,6], Sang-Hyun Oh[5], Hilal A. Lashuel[3], and Hatice Altug[1,*]

[1] Institute of BioEngineering, École Polytechnique Fédérale de Lausanne (EPFL), Lausanne 1015, Switzerland

[2] ICFO– Institut de Ciències Fotòniques, The Barcelona Institute of Science and Technology, 08860 Castelldefels, Spain

[3] Laboratory of Molecular and Chemical Biology of Neurodegeneration, Brain Mind Institute, Faculty of Life Sciences, École Polytechnique Fédérale de Lausanne (EPFL), Lausanne 1015, Switzerland

[4] School of Chemistry, Joseph Black Building, University of Glasgow, Glasgow, G12 8QQ, United Kingdom

[5] Department of Electrical and Computer Engineering, University of Minnesota, 55455 Minnesota, USA

[6] Department of Chemistry, Lehigh University, Bethlehem, Pennsylvania, 18015, USA

[§] These authors contributed equally.

*e-mail: hatice.altug@epfl.ch

Supplementary Note 1: Generalized metasurface with three resonances

The multi-resonance metasurface can be generalized to incorporate additional resonances. In Fig. 1 we show the metasurface design that accommodates three resonances. The multi-resonant metasurface is composed of three sub-arrays of nano-antennas whose periodicities are in a 1:2:4 ratio. The experimental reflection spectrum shows three well-defined optical resonances. This technique can be further extended to accommodate a larger number of resonances by incorporating additional sub-arrays.

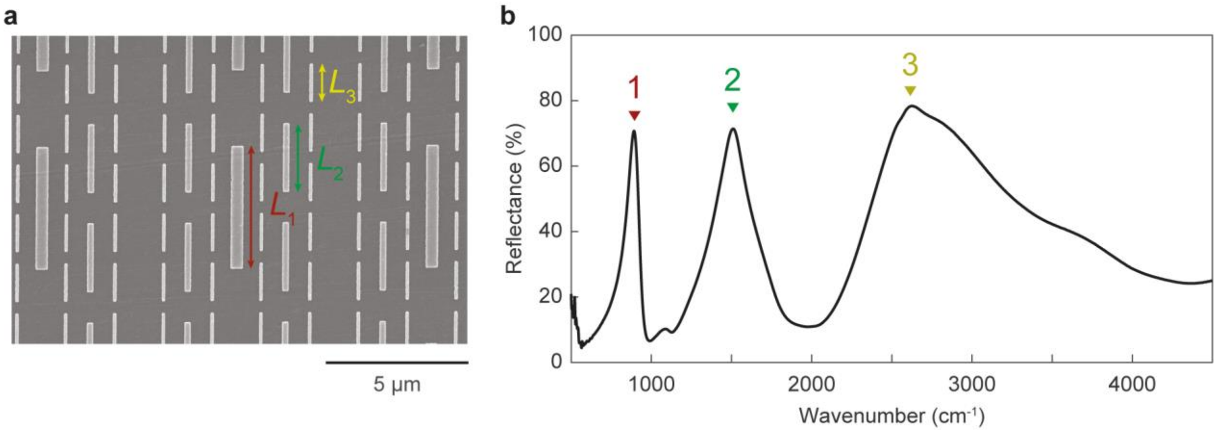

Supplementary Figure 1. Generalized metasurface. **a** Scanning electron microscope image of the generalized metasurface that accommodates three resonance peaks. The metasurface is composed of three sub-arrays of nanodipoles with lengths $L_1 = 4.10$, $L_2 = 2.30$, $L_3 = 1.27$, widths $W_1 = 0.4$, $W_2 = 0.2$, $W_3 = 0.1$ and periodicities $P_1 = 6.64$, $P_2 = 3.32$, $P_3 = 1.66$ (units in μm). **b** Experimental reflectance spectrum of the generalized metasurface. The reflection spectrum shows three well-defined resonances. Each optical resonances is excited by the corresponding sub-array of nano-dipoles.

Supplementary Note 2: Influence of water absorption in the infrared on metasurface transmission

Microfluidic measurements in the mid-infrared are impacted by strong molecular absorption from the required aqueous buffer solutions close to the target amide and methylene bands. To quantify the influence of water absorption on the spectral shape of our multi-resonant metasurface, we extend the numerical simulations presented in the manuscript by including the full complex refractive index of water in this range. Specifically, we consider the metasurface designs from Fig. 2b of the main text. We compare the reflection spectrum of the designs considering a non-dispersive water environment (Supplementary Fig. 2a) and a water environment with a frequency-dispersive complex refractive index from tabulated data taken from literature (Supplementary Fig. 2b).[1] In both cases the simulated reflection spectrum of the multi-resonant structure shows two peaks at 1.600 and 3.000 cm^{-1}, respectively) that are individually tuned modifying the respective dipole lengths L_1 and L_2. Due to the effect of water absorption bands in this spectral range, the two resonance peaks in the second case show superimposed adsorption bands from water.

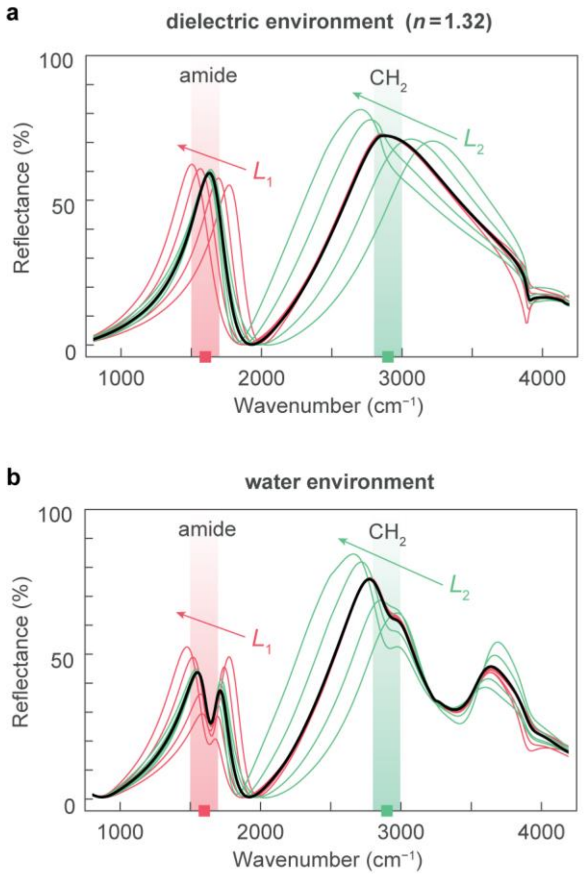

Supplementary Figure 2. Simulated reflectance spectrum of the multi-resonant metasurface for the nominal design (black curve), and with varying lengths L_1 (red curves) and L_2 (green curves) in a ±10% range. The simulated environment is represented in **(a)** as a non-dispersive aqueous medium with refractive index n=1.32. The simulated environment in **(b)** as an absorptive water layer with a frequency-dispersive complex refractive index from tabulated data.

Supplementary Note 3: Near-field penetration depth of the metasurface

To evaluate the penetration depth of the near-field intensity away from the multi-resonant metasurface we have simulated the near-field in the vicinity of the metallic nano-antennas for the two bands of interest. The penetration depth of the metasurface is δ=5nm for the methylene band and δ=10nm for the amide band (for 1/e drop in intensity). As expected, the penetration depth for the amide band is higher than for the methylene band due to longer wavelengths. Of course, the near-field extends beyond the penetration depth at a lower intensity and, for instance, the metasurface can access up to a 20nm depth with 10% of the near-field peak intensity. This result contrasts with the 1-2nm depth achieved in SERS, the Raman counterpart of SEIRA, and allows the metasurface to probe multiple molecules in multi-layers systems (Fig. 3) and the molecules inside the vesicles in the cargo-release experiments (Fig. 4b).

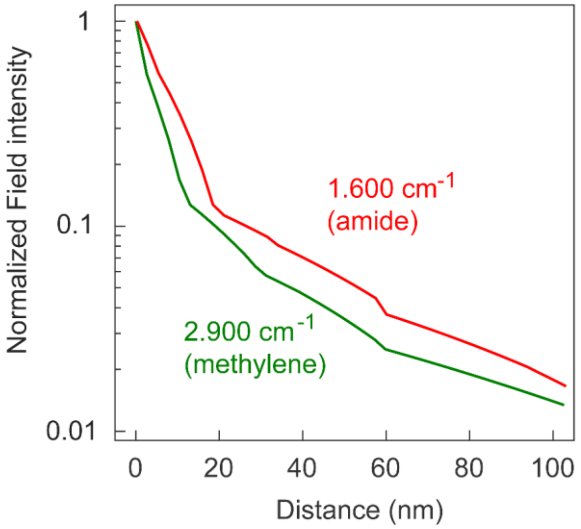

Supplementary Figure 3. Simulated near-field intensity for the multi-resonant metasurface as a function of the distance to the surface of the corresponding nano-antennas. The near-field intensity normalized to its maximum value is shown for the amid band (1.600 cm^{-1}) and methylene band (2.900 cm^{-1}).

Supplementary Note 4: Verification of membrane fluidity via FRAP measurements

To assess the fluidity of the supported lipid bilayers used in our real-time microfluidic experiments, we perform fluorescence recovery after photobleaching (FRAP) measurements. The FRAP experiments shown in Supplementary Fig. 4 were carried out with a Zeiss LSM 710 upright confocal microscope following the protocol from Ref.[2], but with NBD-PC (1-palmitoyl-2-{12-[(7-nitro-2-1,3-benzoxadiazol-4-yl)amino]dodecanoyl}-sn-glycero-3-phosphocholine, Avanti Polar Lipids, Inc.) lipids instead of Texas Red DHPE (1,2-dihexadecanoyl-sn-glycero-3-phosphoethanolamine, Life Technologies).

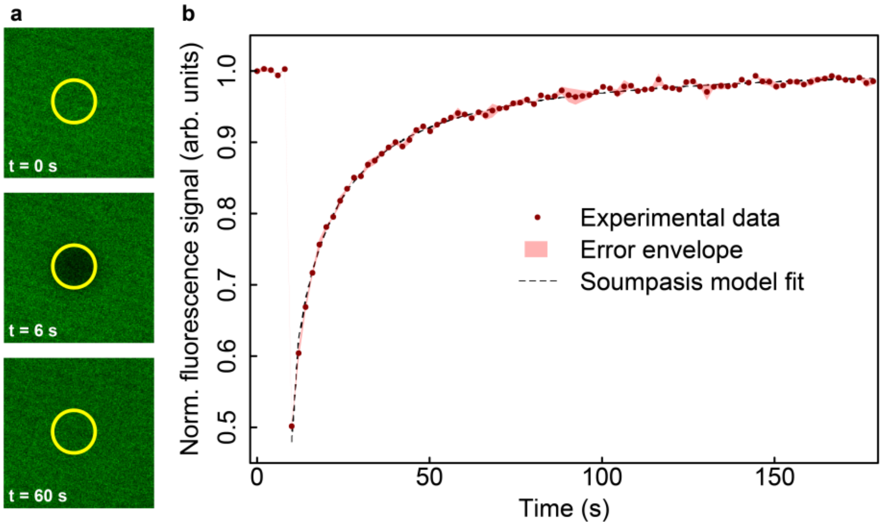

Supplementary Figure 4. Membrane fluidity. **a** FRAP microscopy images of a fluorescently tagged supported lipid bilayer at measurement start (t = 0 s), directly after bleaching (t = 6 s) and at t = 60 s. The bleaching spots have a diameter of 10 µm and are indicated with yellow circles. **b** Data analysis of FRAP measurements confirm the fluidity of the lipid membranes utilized in our experiments. The average of three measurements as well as the error envelope (standard error of the mean) is shown.

Data analysis is performed with the FRAP Analyzer program (University of Luxembourg; http://actinsim.uni.lu) as described previously.[2] After photobleaching, we observe a fast recovery of the fluorescence signal on a timescale of 50 s, which confirms the fluidity of the bilayers utilized in our experiments and is consistent with previous studies on similar membrane systems[2].

Supplementary Note 5: Determination of supported lipid bilayer thickness

The thickness of the supported lipid membranes in our experiments is determined via a multi-parametric surface plasmon resonance (SPR) spectroscopy recording angle-scanning SPR data simultaneously at three different wavelengths (Bionavis 210A VASA). In particular, we perform the SLB formation using DOPC vesicles prepared in the same way as for the other SLB experiments and inject them at the same flow rate over gold chips coated with ALD-deposited silica.

The resulting SPR signal curves before and after formation of a SLB (labelled baseline and membrane, respectively) are shown in Supplementary Fig. 5. The formation of the membrane induces a clear shift of the resonance angle for all three individual laser wavelengths. To extract the thickness of the lipid membrane, we fit a dielectric multilayer model to the measured SPR curves using BioNavis' LayerSolver software. The fitting procedure works by modeling the multilayered optical system in terms of thickness, refractive index and attenuation coefficient, and then numerical calculations are iteratively performed using Fresnel's equations and a transfer matrix formalism of 2 × 2 matrices[3].

The parameters of the multilayer model for the baseline and membrane cases are shown in Supplementary Tables 1 and 2, respectively. The calculations yield a refractive index of n=1.45 and a thickness of 5.1 nm for the SLB. Both values agree very well with values reported in the literature[4,5], and confirm the presence of a *single* DOPC bilayer on the surface. It is important to note that our multi-parametric SPR results provide a thickness of 10.48 nm for the SiO2 which is matching to the nominally used value in ALD deposition. This also evidences that the thickness readings provided by SPR are reliable.

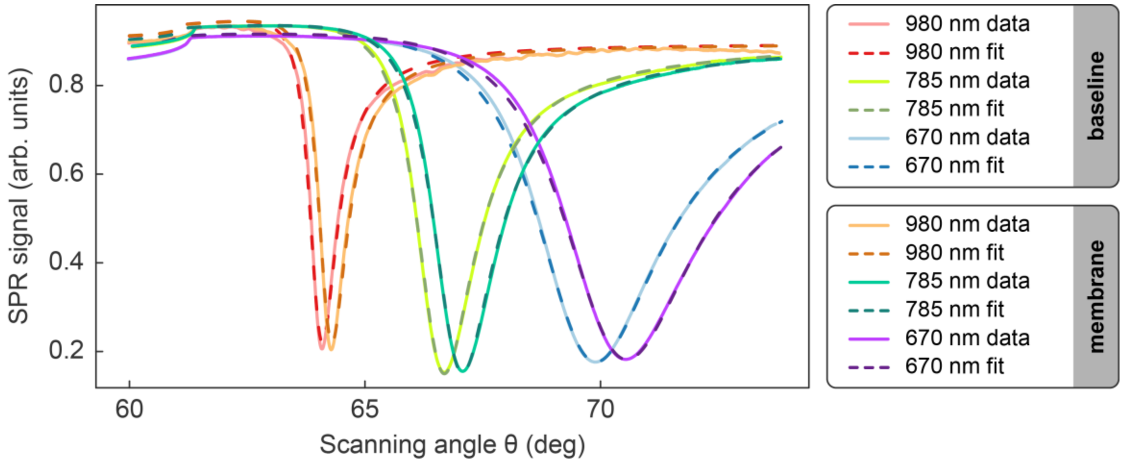

Supplementary Figure 5. SPR measurement for confirming membrane thickness. Angle-scanning SPR signal curves for the three individual laser sources with multi-parametric Bionavis 210A VASA SPR instrument. The formation of the membrane induces a clear shift of the resonance angle in all three measurement channels.

Layer		Support	Au	SiO_2	PBS
n	670 nm	1.5202	0.17215	1.43351	1.33011
	785 nm	1.5162	0.19226	1.45443	1.32887
	980 nm	1.5129	0.26653	1.41842	1.32533
k	670 nm	0	3.86192	0.00213	0.00178
	785 nm	0	4.78515	0.00633	0.00011
	980 nm	0	6.22052	0	0
d (nm)		∞	57.01	10.48	∞

Supplementary Table 1 Model parameters from the baseline measurement (without membrane) establish the properties of the multilayer system. Obtained values show fair agreement with tabulated values for the constituent material components. Values on grey shaded cells indicate parameters which were fixed, and values on orange shaded cells indicate parameters which were set as variables in the calculations.

Layer		Support	Au	SiO_2	DOPC	PBS
n	670 nm	1.5202	0.17215	1.43351	1.45068	1.33011
	785 nm	1.5162	0.19226	1.45443	1.45169	1.32887
	980 nm	1.5129	0.26653	1.41842	1.451	1.32533
k	670 nm	0	3.86192	0.00213	0.00578	0.00178
	785 nm	0	4.78515	0.00633	0.00208	0.00011
	980 nm	0	6.22052	0	0	0
d (nm)		∞	57.01	10.48	**5.07**	∞

Supplementary Table 2 SPR multilayer model fitting results, membrane. Model parameters for a fully formed supported lipid membrane on the SPR chip. A membrane thickness of 5.07 nm is obtained, indicating the presence of a *single* bilayer membrane on the surface. Values in grey shaded cells indicate parameters which were fixed, and values on orange shaded cells indicate parameters which were set as variables in the calculations.

Supplementary Note 6: Regression signals for a variable surface density of streptavidin molecules

We have replicated the lipid-streptavidin binding experiment in Fig. 3 for a variable density of streptavidin molecules in the surface. This density has been controlled by modifying the concentration of biotinylated lipid in the membrane, which corresponds to the density of binding site available for streptavidin. As observed in Supplementary Fig. 6, the levels reached by the streptavidin regression signals vary accordingly to the biotinylated lipid concentration in the membrane. For a biotinylated lipid concentration of 0%, 1% and 5%, the streptavidin signal values are 0.0, 0.2 and 1.0, demonstrating the direct correlation between streptavidin amount and regression signal. For a streptavidin monolayer, the density of molecules on the surface has been approximated as $5*10^{12}$ molecules·cm^{-2}.[6] Consequently, we can estimate the amount of bound streptavidin molecules in our experiments as 0, $1*10^{12}$ and $5*10^{12}$ molecules·cm^{-2} for the given biotinylated lipid amounts of 0%, 1% and 5%. This result illustrates that the regression signals are able to capture the number of molecules in the sensor surface relative to the density of molecules in the experiment done to obtain the reference spectrum for the same analyte.

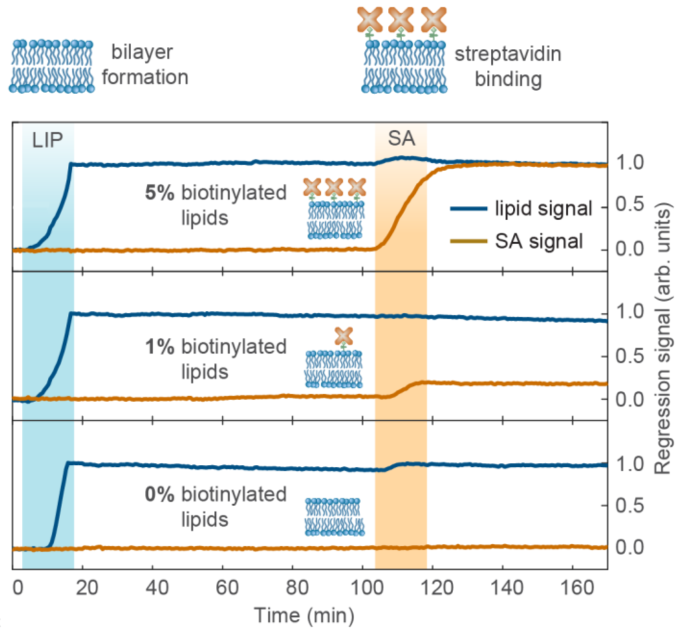

Supplementary Figure 6. Regression signals for a variable surface density of streptavidin molecules.

Supplementary Note 7: Principal Component Analysis

The linear regression analysis in the manuscript decomposes the measured time-resolved absorbance spectra into a linear combination of the two reference absorbance spectra for lipid and protein (see, e.g., Fig. 3 in the main text). The linear regression results are valid as long as the infrared absorption spectrum of the heterogeneous sample is the addition of the absorption of its individual constituents, a condition that is satisfied in all our experiments and analyte concentrations used.

To confirm that two reference spectra used in the experiment corresponding to Fig. 3 are sufficient to capture the full biological information contained in our time-dependent measurements, we performed principal component analysis (PCA) over the spectral data for all the time points in the experiment. In the PCA analysis the time-dependent spectra are decomposed into orthogonal spectra (principal components) maximizing the variance for the first principal component (PC1) and iteratively for the next components (PC2, PC3…). In Supplementary Fig. 7a we see that the first two principal components account for 99.7% of the total variance contained in the data, indicating that there are two linearly independent components in our spectral data (lipid and streptavidin). In Supplementary Fig. 7b we show the PCA scores representing the time evolution of the experiment. There are three regions in the principal component space where the data points accumulate, which correspond to (a) the start of the experiment with no molecules adsorbed, (b) the lipid membrane and (c) the lipid membrane with immobilized streptavidin. The data points linking these three regions correspond to the transients when the lipid bilayer is being formed and during the streptavidin binding. The interpretation of principal components is not straightforward in general, however in this case it is clear that the first principal component (PC1) corresponds mainly to the streptavidin spectrum and the second principal component (PC2) corresponds mainly to the lipid spectrum. Additionally, the large variance explained by PC1 (>80%) is due to the fact that the lipid injection took place at the beginning of the experiment and only a small fraction of the time points are free of lipids, leaving a relatively small variance for PC2 (lipid).

In addition, we compared the measured spectrum (dashed lines) and the linear regression result (solid lines) in the relevant amide and methylene bands. As an example, we show in Supplementary Fig. 7c the comparison for a specific time point after the lipid and streptavidin binding. Agreement between the data sets is excellent, validating the suitability of utilizing a linear regression for analyzing our experimental data. This agreement is not specific to the chosen time point and all other time points show similar agreement between measured data and linear regression results.

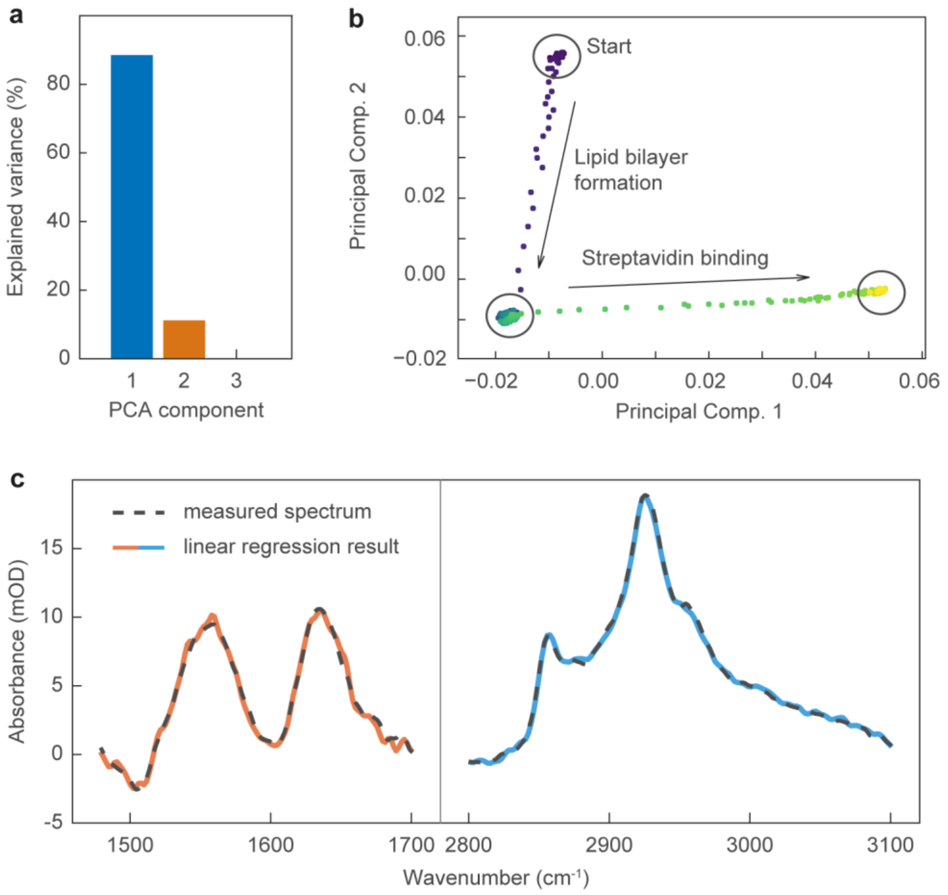

Supplementary Figure 7. Validation of linear regression approach. **a**, PCA explained variance for the time-resolved absorbance spectra in Fig. 3e of the main text, which confirm the presence of two independent components in the experimental data set. **b**, PCA scores for the main two principal components obtained from the spectral data over all the time points show the lipid bilayer formation and the melittin binding. **c**,The measured experimental absorbance spectrum (dashed lines) is in excellent agreement with the linear regression result (solid lines) based on the two reference spectra for lipid and protein.

Supplementary Note 8: Bulk circular dichroism measurements of lipid/melittin association

To validate the association of the melittin used in our experiments to the lipid membrane, we performed circular dichroism (CD) measurement in bulk solution. CD spectra were recorded on a Jasco J-815 CD spectrometer operated at 20°C, and acquired from 195 nm to 250 nm at a scan rate of 50 nm·min^{-1} and in increments of 0.2 nm. For each sample, five spectra are averaged and smoothed using binomial approximation. The melittin and vesicles concentrations are 20 μM and 1 mg·ml^{-1}, respectively.

Bulk CD results in Supplementary Fig. 8 clearly show that the random coil secondary structure of melittin changes into a α-helical conformation in the presence of DOPC lipid vesicles, confirming the efficient association of melittin to the lipid membrane observed in our microfluidic measurements.

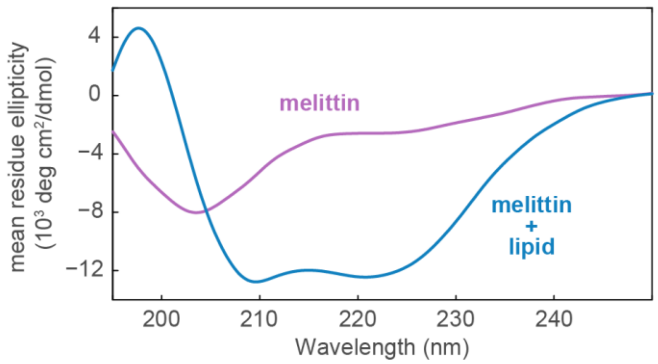

Supplementary Figure 8. Bulk circular dichroism measurements. CD spectra of a melittin solution before (purple line) and after (blue line) the addition of DOPC vesicles clearly show a strong conformational change of the melittin secondary structure, confirming its efficient association with the lipid membrane.

Supplementary Note 9: Fluorescence validation of melittin-induced lipid membrane disruption

To confirm the displacement of lipids in the bilayer membrane as a consequence of the injection of melittin (Fig. 4a of the main text) we reproduced the experiment with fluorescently-tagged lipids. To ensure consistency with the presented FTIR results, the same microfluidic setup of PDMS cell and syringe pump is used. The experiments start with the formation of supported lipid bilayer with fluorescence tagged lipids (Texas Red 1,2-dihexadecanoyl-sn-glycero-3-phosphoethanolamine) on the SiO_2 coated substrates. Continuous fluorescence images are recorded during bilayer formation and subsequent melittin-induced disruption using a confocal laser-scanning microscope (LSM 700, Zeiss Microscopy) with a 10x objective and analyzed using the Zen software (Zeiss Microscopy). The fluorescence signal is obtained by integrating the fluorescence image over a square region corresponding to an area of approximately 100 µm by 100 µm on the chip.

We observed that the arrival of melittin molecules at 100 µM concentration induces a decrease of the fluorescent signal from lipid molecules. The decrease of fluorescent signal is approximately 50%, which is consistent with the SEIRA signals in Fig. 4a. This strong decrease of the lipid fluorescence contrast to the relatively flat fluorescent signal measured in the absence of melittin.

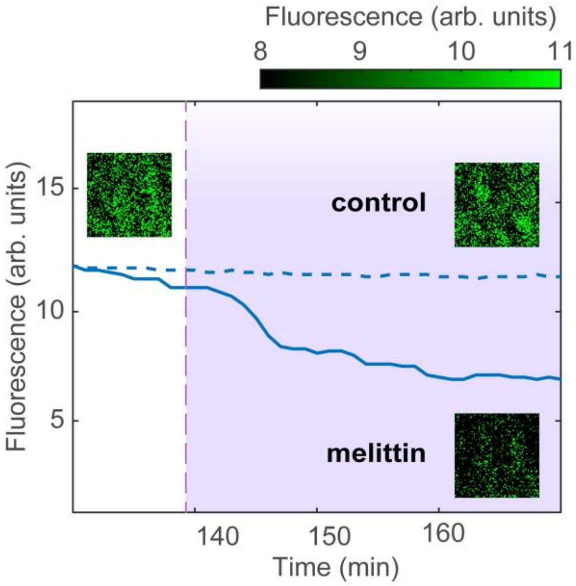

Supplementary Figure 9. The melittin-induced disruption of the lipid membrane is further validated using a fluorescently tagged lipid membrane. The decrease in fluorescence signal level produced after the injection of melittin at 100 µM concentration indicates a displacement of lipid molecules upon melittin association, while the control with no melittin results in a flat fluorescence signal.

Supplementary Note 10: Linear regression reference spectra for vesicular cargo release experiment

The vesicular cargo release experiment in Fig. 4b of the main text includes three significant molecular components: lipid vesicles, melittin, and GABA cargo. The corresponding reference spectra for use in the linear regression analysis were acquired during independent measurements and are shown in Supplementary Fig. 10. Significantly, the three reference spectra form a linearly independent set, allowing for the efficient differentiation of the compounds using linear regression analysis.

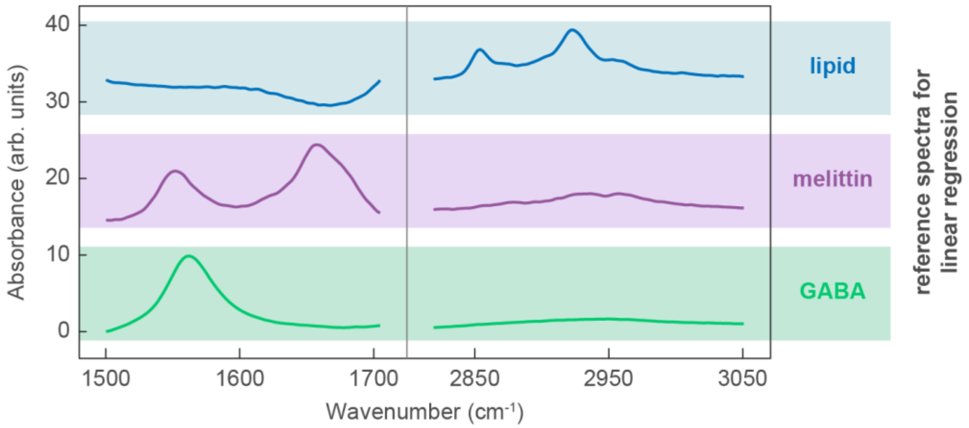

Supplementary Figure 10. Reference spectra for the vesicular cargo release experiment. Reference spectra for the three significant biological components are acquired in separate experiments and demonstrate sufficient linear independence for linear-regression-based chemical discrimination.

Supplementary Note 11: GABA sensing on functionalized metasurface

To confirm that the stable GABA regression signal in Fig. 4b of the main text originates from GABA molecules encapsulated in the lipid vesicles and not from simple adsorption to the sensor metasurface, we flow a large concentration (100 mg·mL^{-1}) of GABA over our sensor metasurface without the presence of any lipid vesicles (Supplementary Fig. 11). We observe a strong peak of the GABA regression signal, which disappears completely through continuous washing with running buffer solution. These results show that GABA does not readily adsorb on our sensor metasurface and thus validates that we indeed observe encapsulated GABA molecules in our synaptic vesicle mimic experiment in Fig. 4b of the main text.

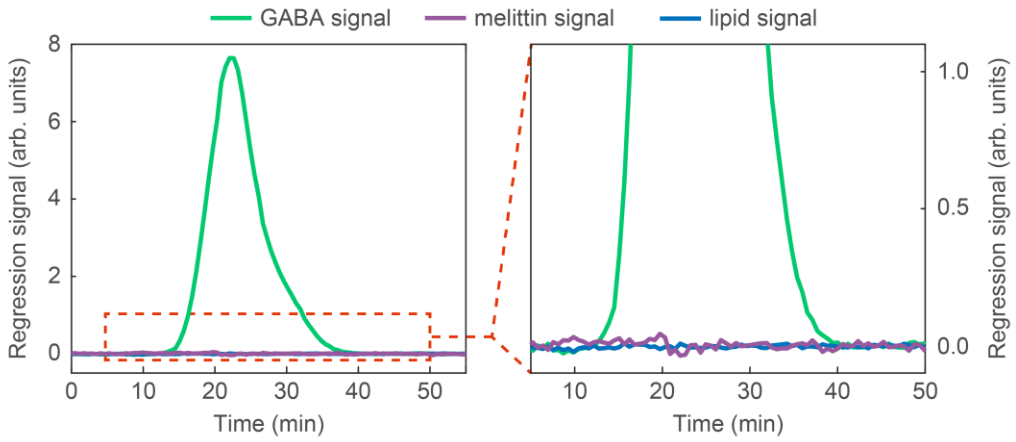

Supplementary Figure 11. GABA adsorption directly on functionalized metasurface. The control experiment is performed by injecting GABA directly on top of the funtionalized metasurface as described in Fig. 5 of the main text, without encapsulating it in vesicles. We observe a strong peak of the GABA regression signal, which disappears completely through continuous washing with running buffer solution. This behaviour confirms that GABA molecules do not adsorb on the sensor metasurface. For easier comparison, the scale in the right panel is chosen to match the scale in Fig. 4c of the main text.

Supplementary References

1. Hale, G. M. & Querry, M. R. Optical Constants of Water in the 200-nm to 200-µm Wavelength Region. *Appl. Opt.* **12,** 555 (1973).
2. Limaj, O. *et al.* Infrared Plasmonic Biosensor for Real-Time and Label-Free Monitoring of Lipid Membranes. *Nano Lett.* **16,** 1502–1508 (2016).
3. Kari, O. K. *et al.* Multi-parametric surface plasmon resonance platform for studying liposome-serum interactions and protein corona formation. *Drug Deliv. Transl. Res.* **7,** 228–240 (2017).
4. Cho, N. J. *et al.* Alpha-helical peptide-induced vesicle rupture revealing new insight into the vesicle fusion process as monitored in situ by quartz crystal microbalance-dissipation and reflectometry. *Anal. Chem.* **81,** 4752–4761 (2009).
5. Attwood, S. J., Choi, Y. & Leonenko, Z. Preparation of DOPC and DPPC supported planar lipid bilayers for atomic force microscopy and atomic force spectroscopy. *Int. J. Mol. Sci.* **14,** 3514–3539 (2013).
6. Reiter, R., Motschmann, H. & Knoll, W. Ellipsometric characterization of streptavidin binding to biotin-functionalized lipid monolayers at the water/air interface. *Langmuir* **9,** 2430–2435 (1993).
